# Successful Repair With Strategic Omentectomy and Meticulous Suturing for Incarcerated Massive Umbilical Hernia in an Adult: A Case Report

**DOI:** 10.7759/cureus.53929

**Published:** 2024-02-09

**Authors:** Toru Zuiki, Jun Ohki, Takashi Ui

**Affiliations:** 1 Surgery, Yuki Hospital, Yuki, JPN

**Keywords:** far-near/near-far stitch, obesity, incarceration, suture repair, umbilical hernia

## Abstract

This case report details the successful management of a massive incarcerated umbilical hernia in an obese adult patient. Strategic integration of omentectomy and meticulous suturing, excluding mesh repair due to comorbidities of obesity and poorly controlled diabetes, led to an uneventful postoperative course. The 65-year-old female underwent semi-emergency surgery, involving the repositioning of the incarcerated intestinal tract into the abdominal cavity through a substantial omentectomy. Closure of the hernia orifice was performed utilizing alternating absorbable interrupted sutures and non-absorbable far-near/near-far stitches. A myofascial release incision in the bilateral rectus abdominis muscle's anterior sheath further contributed to the procedural success. A postoperative computed tomography (CT) scan confirmed no abdominal wall dehiscence. This case highlights the effectiveness of tailored surgical procedures and provides insights into the management of adult umbilical hernias with complex clinical comorbidities.

## Introduction

The European Hernia Society (EHS) defines an umbilical hernia as one located from 3 cm above to 3 cm below the umbilicus. The EHS classifies hernias based on their size [1]. Clinical considerations for adult umbilical hernias involve determining optimal surgery timing due to the high incarceration risk, selecting the appropriate repair procedure, and preventing recurrence. We present a case of a massive umbilical hernia in an adult female that expanded over time, leading to prolapse and subsequent intestinal obstruction, necessitating semi-emergency surgery.

## Case presentation

**Figure 1:**
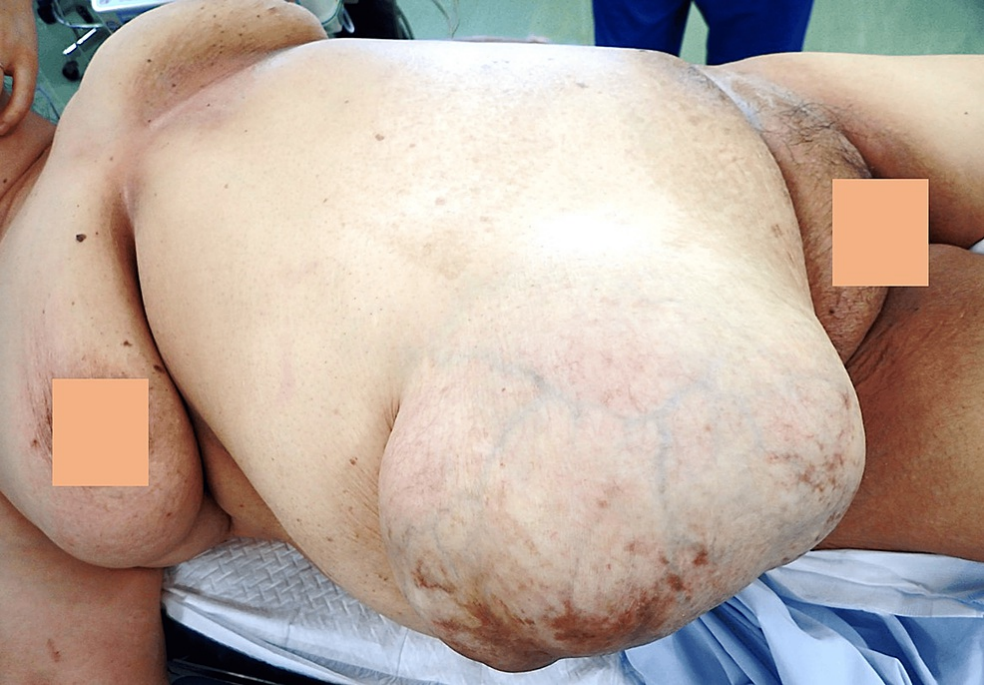
Patient with an incarcerated massive umbilical hernia in the supine position

We present a 65-year-old female with a ventral hernia without a bandage for over 10 years, diagnosed as an umbilical hernia via computed tomography (CT) scan. She exhibited no improvement in diabetes mellitus and obesity despite treatment, due to poor medical compliance and dietary failure. Her body weight remained constant, delaying surgical management of the hernia for years. She was admitted with vomiting and abdominal pain due to the incarcerated hernia (Figure 1).

**Figure 2:**
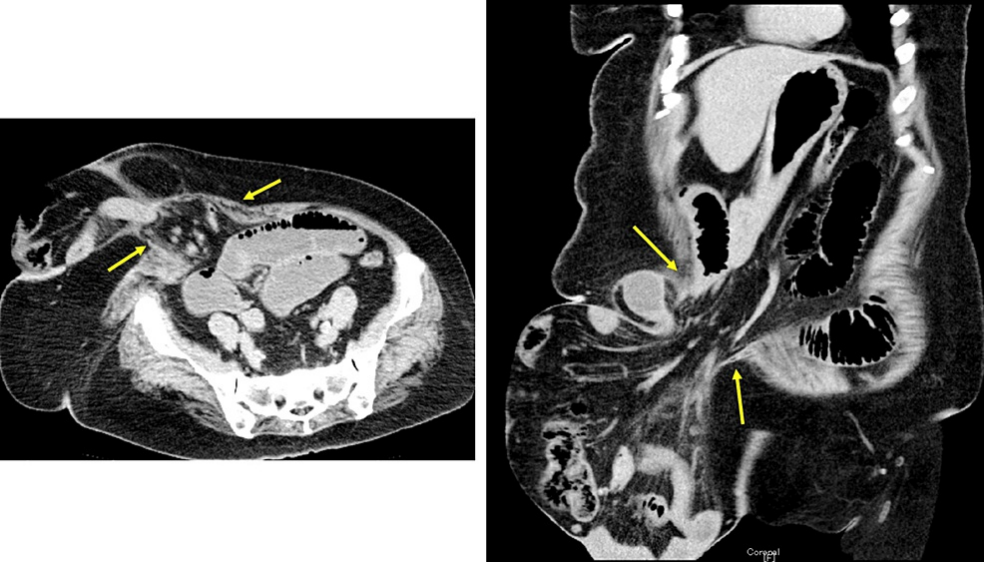
CT scan findings in axial plane (left) and coronal plane (right) CT scan showed herniation of the terminal ileum and ascending colon in the umbilical hernia, with small intestine dilation oral to the hernia. The hernia orifice measured 8 cm (arrow). CT: computed tomography

The hernia was 25 cm in diameter. She weighed 78 kg with a height of 154 cm, yielding a body mass index (BMI) of 32.9 kg/m². CT scan showed herniation of the terminal ileum and ascending colon in the umbilical hernia, with dilation of the proximal small intestine. The hernia orifice measured 8 cm (Figure 2).

After small intestine decompression using a long ileus tube, hernia surgery was performed.

**Figure 3:**
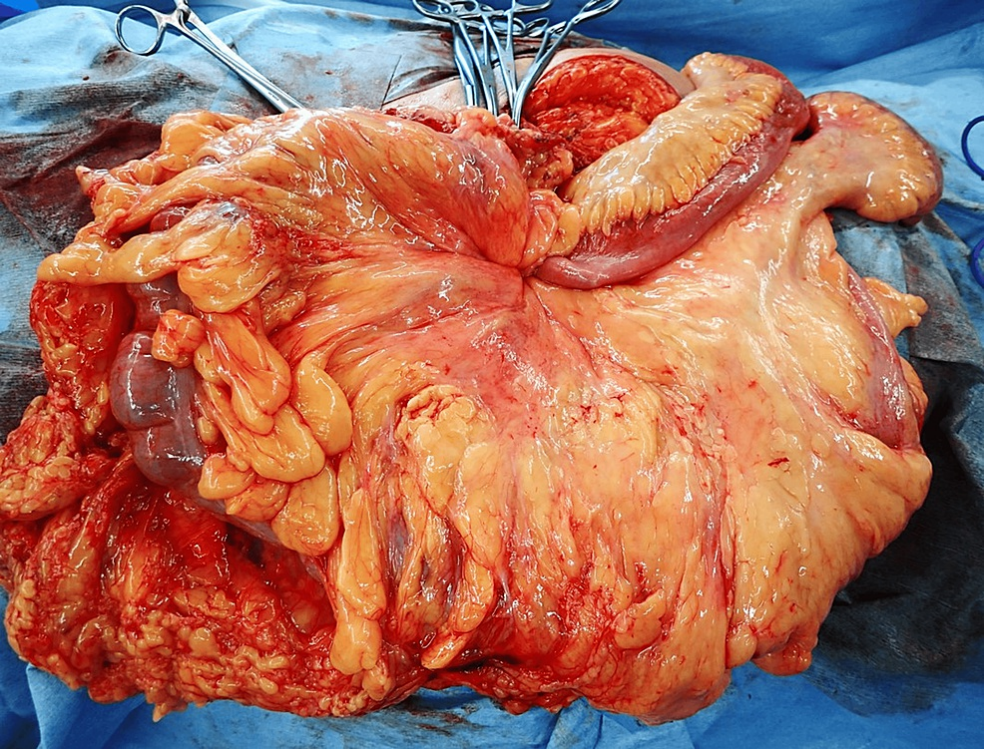
Intraoperative findings after hernia sac opening Opening the sac revealed approximately 50 cm of the terminal ileum, cecum, and ascending colon, all unstrangulated.

During surgery, a lateral incision was made to the left of the hernia. The umbilical hernia sac was exposed, and the linea alba was divided further. Opening the sac revealed approximately 50 cm of the terminal ileum, cecum, and ascending colon, all unstrangulated (Figure 3).

**Figure 4:**
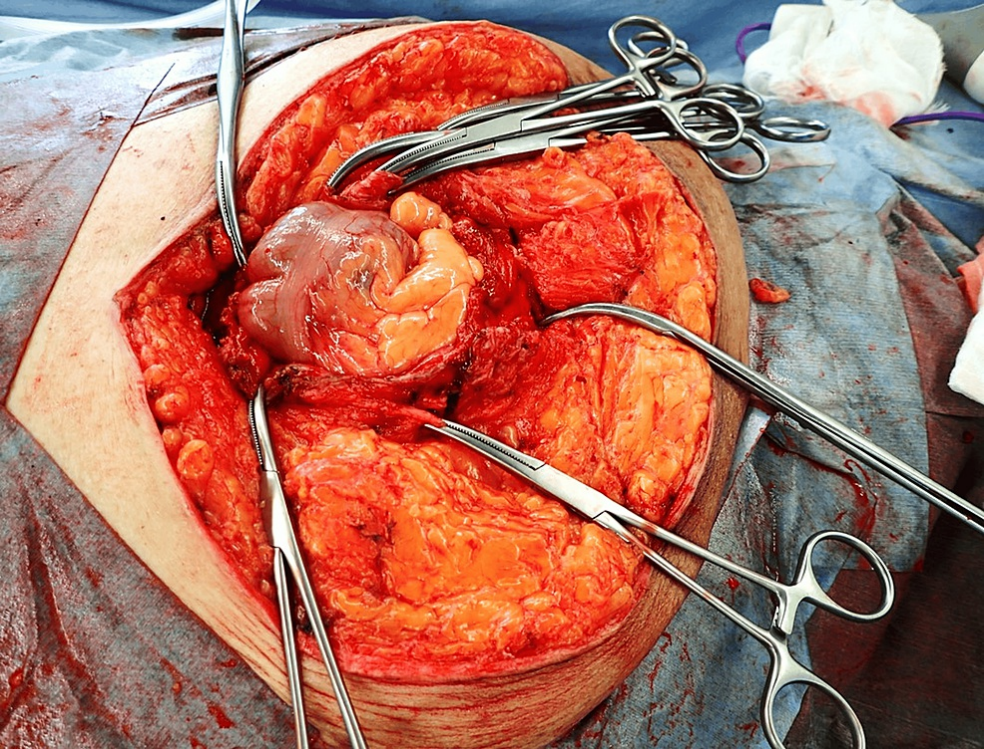
Intraoperative findings after repositioning Incarcerated intestines were all repositioned into the abdominal cavity after omentectomy.

The right omentum part adhered to the hernia sac's peritoneum. A tube introduced into the terminal ileum aspirated intestinal contents. A significant amount of the omentum, adhering to the hernia sac and remaining outside the peritoneum, required resection to reduce intraperitoneal volume. After effective decompression, the terminal ileum and right colon portion were repositioned into the abdominal cavity (Figure 4).

**Figure 5:**
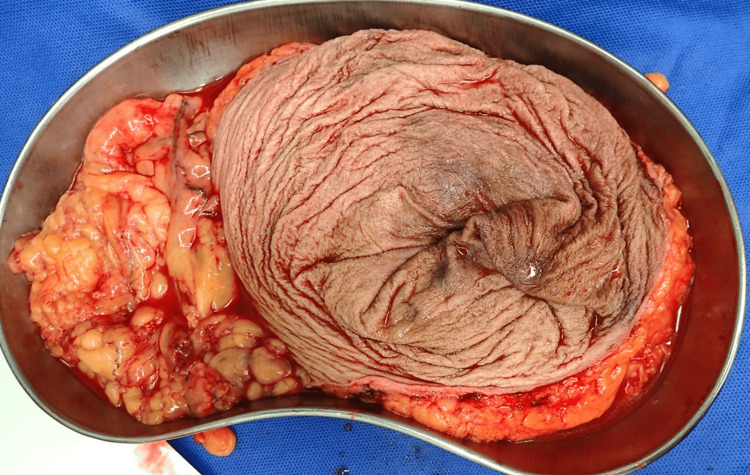
Resected specimen The hernia sac and stretched skin were resected with the adhered omentum.

The hernia sac and stretched skin were resected (Figure 5).

**Figure 6:**
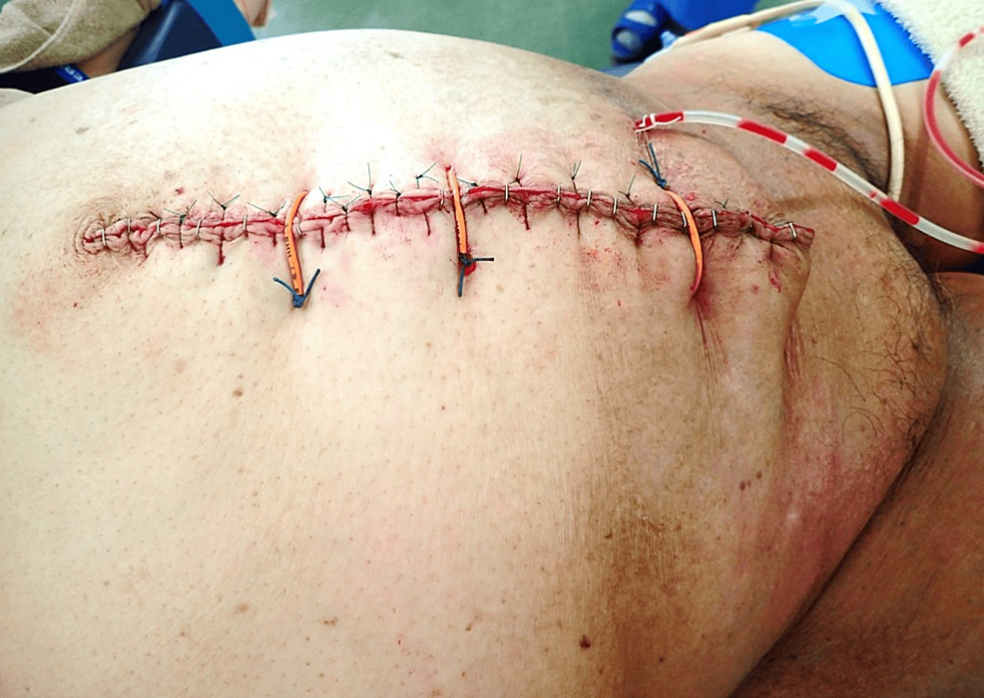
Immediate postoperative image

The abdominal wall was closed without mesh due to infection risk in an uncontrolled diabetic state (HbA1c: 9.6%). Three full-thickness sutures with a rubber tube were initially placed to withstand abdominal wall tension. The peritoneum was closed with continuous sutures, and the fascia was closed using alternating absorbable interrupted sutures and non-absorbable far-near/near-far stitches. A 20 cm myofascial release incision was made in the rectus abdominis muscle's anterior sheath on both sides. The 10 cm thick subcutaneous fat layer, divided into two (including Scarpa's fascia in the superficial layer), was sutured with absorbable sutures to prevent dead space formation. A subcutaneous closed suction drain was placed (Figure 6).

**Figure 7:**
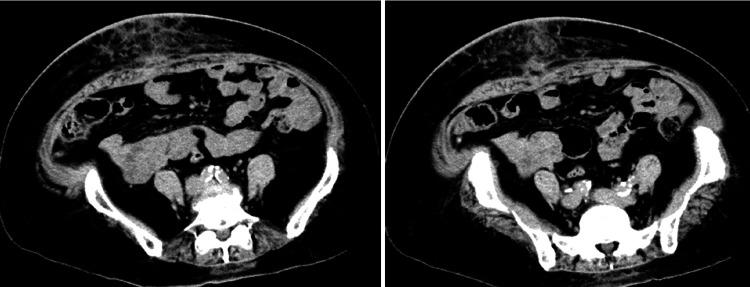
Postoperative CT scan findings A 45-day postoperative CT scan showed no abdominal wall dehiscence. CT: computed tomography

The postoperative course was uneventful, and a 45-day postoperative CT scan showed no abdominal wall dehiscence (Figure 7). The patient has had no recurrence of abdominal wound hernia for 30 months postoperatively.

## Discussion

Repairing this patient's incarcerated umbilical hernia highlighted two crucial surgical aspects. Extensive omentectomy enabled the incarcerated intestine's reduction into the abdominal cavity without causing excessive intraperitoneal compression. A specialized suturing method for abdominal wall repair without mesh prevented postoperative wound dehiscence.

The risks of incarceration and strangulation are well-documented in adult umbilical hernias. Strangulation risk is estimated at up to 17%, three times higher than in femoral hernias [2]. The International Endohernia Society (IEHS) guidelines state umbilical hernias obstruct five times more often than other ventral and incisional hernias, with emergency repairs associated with high morbidity [3]. Elderly patients face a 3.5-fold higher emergency surgery mortality rate than adults [4]. A notable case involved massive colon gangrene due to a strangulated umbilical hernia [2]. While our patient's incarcerated intestine was not strangulated and was preserved, it could have strangulated without effective long ileus tube decompression.

Although suture repair of umbilical hernias is common, recurrence poses a significant problem. A systematic review identified risk factors for recurrence, including mesh use, defect size, and patient characteristics such as BMI and diabetes [5]. The overlooked presence of a fenestrated linea alba, indicating multiple fascial defects associated with recurrence, was noted in umbilical hernia repair [5].

A recent randomized, double-blind, controlled multicenter trial reported lower recurrence rates in adult umbilical hernia mesh repairs than suture repairs [6]. Updated International Endohernia Society (IEHS) guidelines recommend repairing all abdominal wall defects with prosthetic mesh [7]. Mesh repair, particularly recommended for recurrent hernias, has a higher local complication rate, including surgical site infection [8], seroma, and chronic pain. The IEHS guidelines cite fewer wound infections and complications in laparoscopic ventral and incisional hernia repairs [7]. A BMI exceeding 30 kg/m² increases recurrence risk, and laparoscopic repair is preferred [7]. A recent report suggested that laparoscopic umbilical hernia repair can be safely performed in male patients with abdominal obesity without additional complication risks [9]. In our patient, we did not choose laparoscopic surgery due to technical difficulties and avoided using mesh due to concerns about infection due to poorly controlled diabetes and obesity.

Our patient's adhered omentum to the hernia sac was resected, effectively reducing intraperitoneal fat volume for abdominal wall suturing. Typically, to prevent adhesive intestinal obstruction, the omentum is neatly spread under the wound when closing the abdomen. Therefore, the concept of strategically removing the omentum to reduce intraperitoneal volume for abdominal wall closure is not widely accepted. Although omentectomy is common in malignant tumor surgeries, such as gastric and ovarian cancers, its metabolic benefit in bariatric surgery is disproven [10].

We typically place prosthetic mesh between the rectus abdominis muscle and the posterior sheath as in the Rives-Stoppa procedure for large incisional hernias. Component separation techniques (CSTs) are used for patients unsuitable for mesh repair. The IEHS guidelines recommend CST for fascial closure in contaminated fields when no mesh is used [11]. In this patient, bilateral rectus abdominis muscle sutured through bilateral myofascial release incisions eliminated the need for CST.

To close the patient's rectus abdominis fascia, we used far-near/near-far sutures, often employed for patients with high intra-abdominal pressure. This technique distributes tension evenly, potentially minimizing wound dehiscence and incisional hernia risks [12]. The patient had an uneventful postoperative course with no wound infection or dehiscence, and a six-week postoperative CT scan confirmed no muscle layer dehiscence and a secure abdominal wall. The patient has had no recurrence of abdominal wound hernia for 30 months postoperatively.

## Conclusions

Strategic omentectomy and a specialized suturing procedure effectively repaired a massive incarcerated umbilical hernia in an obese patient. This case highlights the effectiveness of tailored surgical procedures and provides insights into the management of adult umbilical hernias with complex clinical comorbidities.

## References

[1] Muysoms FE, Miserez M, Berrevoet F (2009). Classification of primary and incisional abdominal wall hernias. Hernia.

[2] Baco S, Mitric M (2022). Gangrene of the colon ascendens, colon transversum, and lienal flexure in a massive strangulated umbilical hernia. Cureus.

[3] Bittner R, Bingener-Casey J, Dietz U (2014). Guidelines for laparoscopic treatment of ventral and incisional abdominal wall hernias (International Endohernia Society (IEHS))-part 1. Surg Endosc.

[4] Patel S, Smiley A, Feingold C, Khandehroo B, Kajmolli A, Latifi R (2022). Chances of mortality are 3.5-times greater in elderly patients with umbilical hernia than in adult patients: an analysis of 21,242 patients. Int J Environ Res Public Health.

[5] Mannion J, Hamed MK, Negi R, Johnston A, Bucholc M, Sugrue M (2021). Umbilical hernia repair and recurrence: need for a clinical trial?. BMC Surg.

[6] Kaufmann R, Halm JA, Eker HH (2018). Mesh versus suture repair of umbilical hernia in adults: a randomised, double-blind, controlled, multicentre trial. Lancet.

[7] Bittner R, Bain K, Bansal VK (2019). Update of guidelines for laparoscopic treatment of ventral and incisional abdominal wall hernias (International Endohernia Society (IEHS))-part a. Surg Endosc.

[8] Winsnes A, Haapamäki MM, Gunnarsson U, Strigård K (2016). Surgical outcome of mesh and suture repair in primary umbilical hernia: postoperative complications and recurrence. Hernia.

[9] Al-Mulhim AS, Memon AQ (2022). Laparoscopic umbilical hernia repair in male patients with abdominal obesity. Pak J Med Sci.

[10] Andersson DP, Eriksson-Hogling D, Bäckdahl J (2017). Omentectomy in addition to bariatric surgery-a 5-year follow-up. Obes Surg.

[11] Bittner R, Bain K, Bansal VK (2019). Update of guidelines for laparoscopic treatment of ventral and incisional abdominal wall hernias (International Endohernia Society (IEHS)): part B. Surg Endosc.

[12] Garg S, Yadav MS, Singhal K (2023). A clinical comparative study of rectus sheath closure techniques in emergency exploratory laparotomy: evaluating “far-near-near-far” vs. conventional closure approach. Cureus.

